# Efficient Biofilms Eradication by Enzymatic-Cocktail of Pancreatic Protease Type-I and Bacterial α-Amylase

**DOI:** 10.3390/polym12123032

**Published:** 2020-12-17

**Authors:** Seung-Cheol Jee, Min Kim, Jung-Suk Sung, Avinash A. Kadam

**Affiliations:** 1 Department of Life Science, College of Life Science and Biotechnology, Dongguk University-Seoul, Biomedi Campus, 32 Dongguk-ro, Ilsandong-gu, Goyang-si 10326, Gyeonggi-do, Korea; markjee@naver.com (S.-C.J.); pipikimmin@naver.com (M.K.); sungjs@dongguk.edu (J.-S.S.); 2 Research Institute of Biotechnology and Medical Converged Science, Dongguk University-Seoul, Biomedi Campus, 32 Dongguk-ro, Ilsandong-gu, Goyang-si 10326, Gyeonggi-do, Korea

**Keywords:** protease type-I, α-amylase, anti-biofilm enzymes, biofilm eradication

## Abstract

Removal of biofilms is extremely pivotal in environmental and medicinal fields. Therefore, reporting the new-enzymes and their combinations for dispersal of infectious biofilms can be extremely critical. Herein, for the first time, we accessed the enzyme “protease from bovine pancreas type-I (PtI)” for anti-biofilm properties. We further investigated the anti-biofilm potential of PtI in combination with α-amylase from *Bacillus sp*. (αA). PtI showed a very significant biofilm inhibition effect (86.5%, 88.4%, and 67%) and biofilm prevention effect (66%, 64%, and 70%), against the *E. coli*, *S. aureus*, and MRSA, respectively. However, the new enzyme combination (*Ec*-PtI+αA) exhibited biofilm inhibition effect (78%, 90%, and 93%) and a biofilm prevention effect (44%, 51%, and 77%) against *E. coli*, *S. aureus*, and MRSA, respectively. The studied enzymes were found not to be anti-bacterial against the *E. coli*, *S. aureus*, and MRSA. In summary, the PtI exhibited significant anti-biofilm effects against *S. aureus*, MRSA, and *E. coli*. *Ec*-PtI+αA exhibited enhancement of the anti-biofilm effects against *S. aureus* and MRSA biofilms. Therefore, this study revealed that this *Ec*-PtI+αA enzymatic system can be extremely vital for the treatment of biofilm complications resulting from *E. coli*, *S. aureus*, and MRSA.

## 1. Introduction

Bacteria demonstrates versatile-tactics to infect humans [1]. In acute infections, they promptly spread and proliferate as a planktonic/individual form [2,3]. But, when an infection reaches the persistent or chronic stage, they largely colonize the tissues and other body-surfaces in highly-organized patterns of multicellular-aggregates termed as biofilms [1,4,5]. Moreover, the important strategy adopted by bacteria for survival against anti-microbial materials and hostile environmental conditions is the formation of a rigid biofilm [1,2,6]. The microbes in a biofilm community exhibit advanced antibiotic resistance that can be up to 1000 times higher than the corresponding planktonic micro-organisms [7]. The contamination of the medical device and food packaging surfaces with pathogenic bacteria might lead to the biofilm formation, thereby it can cause serious acute and chronic infections to people [8,9]. Biofilm composed of multi-species are difficult to remove through host defense systems or by the antibiotic treatment [5,6,10]. Therefore, recently it has become imperative to advance various treatment approaches for biofilm eradication. 

Structurally, biofilms are aggregates of micro-organisms encased in extracellular polymeric substances (EPS) [11,12,13]. The EPS matrix is mainly composed of polysaccharides, extracellular-polymeric substances, lipids, proteins, and extracellular DNA (eDNA) [11]. The EPS allows immobilization of cells and retains them nearby, thus permitting for deep interactions, comprising cell to cell communication, and microconsortia formation [11]. Biofilm formation happens in the four main stages: (1) attachment of bacteria to a surface; (2) formation of microcolony; (3) maturation of biofilm; and (4) dispersal of bacterial biofilm [14,15]. In this process the proteins and polysaccharides from EPS play a vital role [14]. Thus, enzymes which can degrade these proteins and polysaccharides are of high importance in biofilm treatment processes [5,16,17,18,19,20]. Enzymatic degradation of EPS induces susceptibility of the microbes to anti-microbial agents. A previous study showed that enzymes induce the anti-biofilm effects that caused the anti-microbial materials to kill the bacteria released from biofilm [21]. Hence, to introduce more enzymes and enzymatic combinations with excellent capacity of EPS removal will be highly encouraging, and it will enhance the efficacy of biofilm infection treatment strategies.

Efficacious removal of complex biofilms needs the usage of multi-enzyme formulations, which are capable of degrading microbial proteins, eDNA, polysaccharides, and quorum-sensing molecules [22]. These include various enzymes such as proteases, amylases, DNAses, β-glucosidases, and lyticases, etc. [5,19,23,24,25] The protease was found to be more effective compared to amylase for eliminating the *Pseudomonas fluorescens* biofilm [26]. Proteases are of many forms and are well-known as they hydrolyze the peptide bonds and degrade the proteins [27]. Protease induced the degradation of biofilm components and destruction of biofilm backbone [22]. Many proteases from several origins are well studied for anti-microbial and anti-biofilm effects. Bovine pancreatic enzymes are an excellent source for the many therapeutic enzymes [28,29,30,31]. However, protease type I (PtI) from the bovine pancreas is still not evaluated for its potential against the biofilms. Looking at the worsening biofilm infection problems and inefficiencies in their treatment, several kinds of protease and their combinations with the other enzymes are highly important [14]. Furthermore, the effect of this important enzyme needs to be evaluated for both the anti-biofilm activities such as “inhibitions of the established biofilm” and “prevention of the biofilm formations” [32]. There is an extreme need of anti-biofilm enzymes which have both of these potentials: biofilm inhibitions and biofilm prevention [5,33,34]. This kind of formulation having both the capacities will help immensely in the available treatment strategies for biofilm infections [33]. Accordingly, the new protease source “PtI” with a combination of α-amylase (αA) for biofilm inhibitions and biofilm prevention of three major bacteria (*Escherichia coli* (*E. coli*), *Staphylococcus aureus* (*S. aureus*), and methicillin-resistant *S. aureus* (MRSA)) can be a vital study. Hence, this study might add an extremely important and valuable source of multi-enzyme combination to dispersal of the biofilms. Therefore, this kind of enzyme cocktail for biofilm removal is a prerequisite to add better solutions in the treatment of biofilms.

Hence, in this study, PtI from a bovine pancreas was accessed in combination with the αA (denoted as; *Ec*-PtI+αA) against the *Escherichia coli* (*E. coli*), *Staphylococcus aureus* (*S. aureus*), and methicillin-resistant *S. aureus* (MRSA) biofilms for anti-biofilm activities such as “inhibition of established biofilms” and “prevention of biofilm formation”. The studied enzymatic combinations were also accessed for the possible anti-microbial properties against *E. coli*, *S. aureus*, and MRSA. 

## 2. Materials and Methods

### 2.1. Microbial Strains

*Escherichia coli* (KCCM 11234; *E. coli*), *Staphylococcus aureus* (KCCM 11335; *S. aureus*), and methicillin-resistant *Staphylococcus aureus* (ATCC 33591; MRSA) were purchased from the Korean Culture Center of Microorganisms (KCCM, Seoul, Korea) and the American Type Culture Collection (ATCC, Manassas, VA, USA). Protease from bovine pancreas Type I (PtI) having 5 U/mg activity, and α-Amylase from *Bacillus* sp. (Powder form) (αA), having 400 U/mg activity, were purchased from Sigma Aldrich, St. Louis, MO, USA. Each bacterium was incubated on a Tryptic Soy Agar plate (TSA; BD, San Jose, CA, USA) at 37 °C overnight. A colony of the bacterium was inoculated in Tryptic Soy Broth (TSB; BD, San Jose, CA, USA) and incubated at 37 °C 150 rpm overnight.

### 2.2. Biofilm Formation and Inhibition Assay

The biofilm formation experiment was referred to by [35]. All bacteria strains were cultured in TSB medium and dispensed into a 6-well plate. OD600 of 1.0 bacteria were seeded and incubated at 37 °C for 24 h. The formed biofilms are washed two times using PBS very carefully without disturbing the biofilm. The inhibition effect of PtI, αA and *Ec*-PtI+αA on biofilms was estimated on developed biofilms in a 6-well plate using Crystal Violet (CV) assay and plate count method (colony forming units/mL or CFUs) [36]. The enzymes were individually PtI (2 U/mL) and αA (2 U/mL), and in combination *Ec*-PtI+αA (2 U/mL each) were treated with each bacterial biofilm formed in a 6-well plate. This plate was incubated for 37 °C for 2 h for the enzymatic treatment. After incubation was completed, the biofilms were washed very carefully two times with PBS and further added with 0.1% CV solution. After having taken a picture of the 6-well plate, 1 mL of ethanol was added to each well, and the absorbance was estimated at 570 nm. The more bacteria remaining in the biofilm, the higher the absorbance of CV at 570 nm [35,36]. This gave the quantitative measure of the biofilm inhibition after the enzyme treatments. Additionally, to confirm the biofilm inhibition assay, the enzyme-treated biofilms in 6 well plates were added with 1 mL of peptone water. The peptone water solution was well mixed with biofilms by micropipette. Then, 0.1 mL of the sample from each well were transferred onto the TSA plates and incubated at 37 °C for 24 h. The more cells remaining in the biofilm, the more CFU will form and vice versa [36]. This gave the quantitative measure of the biofilm inhibition.

### 2.3. Biofilm Prevention Assay

A bacteria strains of *E. coli*, *S. aureus* and MRSA (OD600 of 1.0) was cultured in TSB medium with the PtI (2 U/mL), αA (2 U/mL) and *Ec*-PtI+αA (2 U/mL each enzyme) into a 6-well plate, respectively. Seeded bacteria with enzymes were incubated at 37 °C for 24 h. The formed biofilms in the presence of enzymes were washed two times using PBS and added with a 0.1% CV solution. After taking a picture of the 6-well plate, 1 mL of ethanol was added to each well, and the absorbance was estimated at 570 nm. Additionally, to confirm the biofilm prevention assay, the biofilms formed in presence of the enzymes in 6-well plates was added with 1 mL of peptone water. The peptone water solution was well mixed with biofilms by micropipette. Then, 0.1 mL of the sample from each well were transferred onto the TSA plates and incubated at 37 °C for 24 h.

### 2.4. Antibacterial Assay

The anti-bacterial effect of PtI, αA and *Ec*-PtI+αA was assessed against the *E. coli*, *S. aureus*, and MRSA. Each cultivated bacteria (OD600 of 1.0) was incubated with enzymes: PtI (2 U/mL), αA (2 U/mL) and *Ec*-PtI+αA (2 U/mL each enzyme) in TSB at 37 °C for 2 h. After incubation, the microbial viability level was evaluated using the microbial viability assay ELISA kit (Dojindo, Kumamoto, Japan) according to the manufacturer’s instructions. Moreover, to confirm the anti-bacteria effects, bacteria were seeded on TSA and incubated at 37 °C for 24 h. After that, colony-forming units of each sample were manually counted.

### 2.5. Statistical Analysis

All results were performed using GraphPad Prism version 5.0 (GraphPad Software, Inc., San Diego, CA, USA). All data were expressed as mean ± SEM. Statistical significance was evaluated by one-way and two-way analysis of variance (ANOVA), followed by Turkey’s multiple comparison test at *p* < 0.05.

## 3. Results and Discussion

### 3.1. Biofilm Inhibition Study

**Figure 1:**
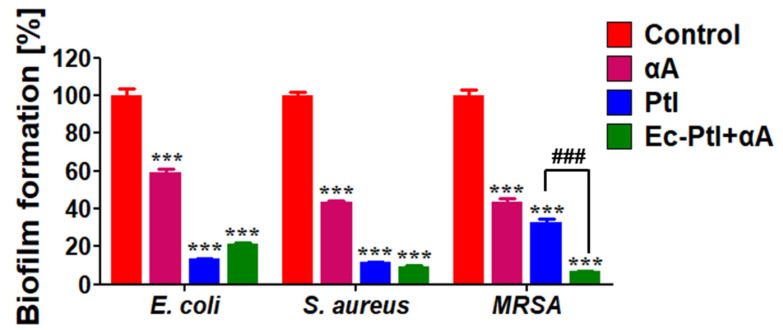
The inhibition effect of PtI, αA, and *Ec*-PtI+αA against biofilm was quantified CV staining at 570 nm. All values are expressed as mean ± SEM (*n* = 3) and significantly different in comparison to controls (***, *p* < 0.001) and to protease (###, *p* < 0.001) by Tukey’s multiple comparison test.

The biofilms of *E. coli*, *S. aureus*, and MRSA were successfully established in 6-well plates. Then, the PtI, αA, and *Ec*-PtI+αA were accessed their potential for biofilm inhibition of *E. coli*, *S. aureus*, and MRSA. The obtained quantitative data for biofilm inhibition of PtI, αA, and *Ec*-PtI+αA were shown in Figure 1. Photographic images of CV staining given in Figure S1 (Supplementary Information). The αA exhibited 40% inhibition of the *E. coli* biofilm. The PtI enzyme acted on the *E. coli* biofilm and gave very significant 86.5% inhibition. The *Ec*-PtI+αA enzyme combination resulted in the 78.6% inhibition of the *E. coli* biofilm. The obtained results conveyed that PtI works far better for *E. coli* biofilm eradication in comparison with the αA and *Ec*-PtI+αA. The role of the enzyme in anti-biofilm results directly depended on the composition and structure of the biofilm [24]. The slightly lower *E. coli* biofilm inhibition by *Ec*-PtI+αA compared to PtI might be due to the interference caused by αA catalytic activity. The obtained result was supported by the previous report Lim et al., 2019 [17]. Lim et al., 2019 [17] studied EPS-protein degradation by proteinase K to control the *E. coli* O157: H7 biofilm efficiently. In this study, PtI acted better in *E. coli* biofilm, mainly due to the efficient degradation of proteins by PtI from the EPS. The obtained biofilm inhibition by enzymatic combinations can be very significant. This information will provide an additional enzymatic source to eradicate the *E. coli* biofilm.

After confirming the anti-biofilm assessments against *E. coli* biofilm, the enzymatic systems were assessed against the *S. aureus* biofilm. The αA, PtI, and *Ec*-PtI+αA were showed 60%, 88.4%, and 90.5% inhibition of the *S. aureus* biofilm, respectively (Figure 1). The photographic images of CV staining were shown in Figure S1 (Supplementary Materials). The PtI and *Ec*-PtI+αA exhibited very high biofilm inhibitions (88.4% and 90.5%) of *S. aureus* biofilm than the αA (60%). Not significant elevation in *S. aureus* biofilm inhibition was observed by *Ec*-PtI+αA than the individual PtI. The culture supernatant of *Pseudomonas aeruginosa* PAO1 containing higher protease activity gave 80% inhibition of *S. aureus* biofilm [37]. Protease aureolysin (Aur) was inhibited 50% of the *S. aureus* biofilm [38]. Protease neutrase from *Bacillus amyloliquefaciens* gave 72% inhibition of the *S. aureus* biofilm [39]. In a comparison of this literature, PtI and *Ec*-PtI+αA exhibited higher biofilm inhibition for *S. aureus* biofilm. Thus, the enzyme PtI and *Ec*-PtI+αA combination can be vital in the treatment of *S. aureus* biofilm.

MRSA is a dangerous pathogen as it encompasses strong resistance against the β-lactam antibiotics [40]. Once the bacteria become resistant to two or more antibiotics, it is usually mentioned as a superbug, multiple-antibiotic-resistant bacteria, or a super-bacterium [41]. Drug-resistant bacteria MRSA (gram-positive) is considered to be a serious threat and is a major challenge to global health [42]. In the biofilm form, MRSA become harder to treat and to handle its consequences [40]. There are limited studies that have investigated the dispersal of MRSA biofilms by applying enzymatic agents [16]. Therefore, in this study, we treated MRSA biofilms with PtI, αA, and *Ec*-PtI+αA. The enzymes; αA, PtI, and *Ec*-PtI+αA showed 60%, 67%, and 93.3% inhibition of the MRSA biofilm (Figure 1). The significant enhancement in the anti-biofilm effect against the MRSA was evident (Figure 1). The EPS composition of the MRSA biofilm might be oriented in such a way that the combination of *Ec*-PtI+αA was able to disperse at a higher rate, rather than the individual enzymes αA and PtI. The obtained anti-biofilm properties of αA, PtI, and *Ec*-PtI+αA against the MRSA biofilm are highly encouraging and could be more suitable for the development of the treatment protocols. It is very important to know the detailed comparison of recently reported proteases for the anti-biofilm effect. The list of recent enzymes applied for biofilm removal was listed in the Table 1. This comparison revealed the newly reported enzyme PtI stand alone, which is worth reporting and is highly efficient at tackling biofilm infections.

Furthermore, the biofilm samples remaining after treatment of enzymes were analyzed for the determination of viable cell numbers by plate count (colony forming units/mL or CFUs) [36,43,44,45]. The enzyme-treated biofilm mixed well with 1 mL of peptone water by using a micropipette to release the cells from the biofilm. The released cells were placed on the Petri dish to confirm the number of cells remaining in biofilms. Figure S2 supplementary information represents the obtained results of cells remained after the enzymatic treatment. The control Petri dishes of *E. coli*, *S. aureus*, and MRSA showed a higher number of colonies. However, the number of colonies was significantly decreased in the PtI- and *Ec*-PtI+αA-treated biofilms. Thus, the obtained results are in agreement with the CV staining quantitative results (Figure 1). Thus, both CV staining and viable cell numbers by plate count results evidenced the eradication of the biofilms. Thus, in summary, PtI was found to be a new protease source and there is a high possibility of the development of PtI-based treatment of biofilms.

### 3.2. Prevention of Biofilm Formation

**Figure 2:**
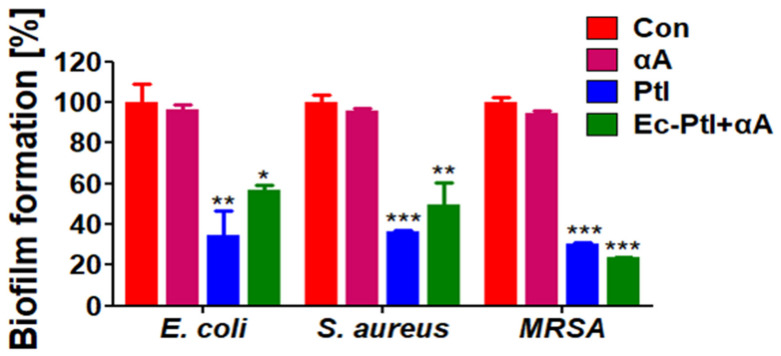
The prevention effect of protease and protease with α-amylase against biofilm formation by *E. coli*, *S. aureus*, and MRSA were performed using quantification of CV staining at 570 nm. All values are expressed as mean ± SEM (*n* = 3) and are significantly different in comparison to controls (*, *p* < 0.05, **, *p* < 0.01, and ***, *p* < 0.001) by Tukey’s multiple comparison test.

**Figure 3:**
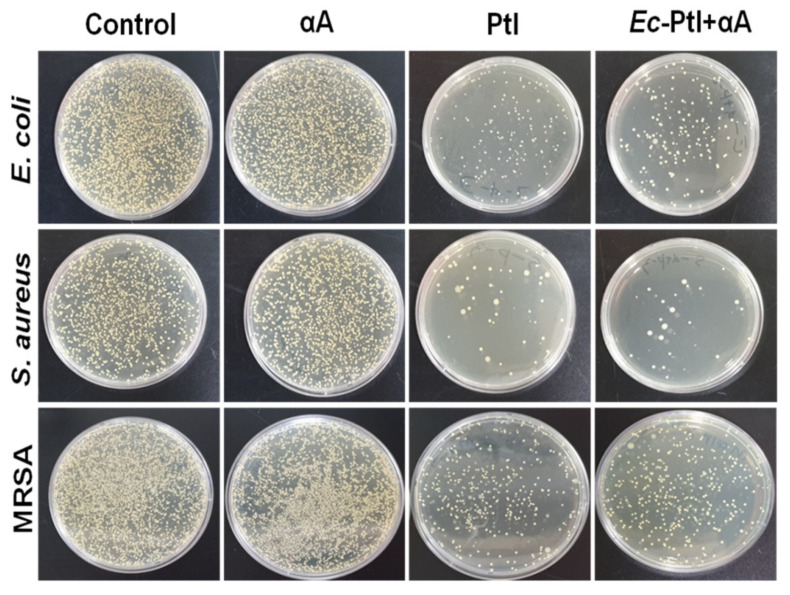
The prevention effect of αA, PtI, and *Ec*-PtI+αA against biofilm formation by *E. coli*, *S. aureus*, and MRSA were performed by colony counting.

After accessing the effect of αA, PtI, and *Ec*-PtI+αA on established biofilms, it is necessary to check if there is any role of these enzymes in the prevention of biofilm formation. Hence, to confirm the biofilm-prevention effect, we inoculated the enzymes αA, PtI, and *Ec*-PtI+αA in a growth medium with bacteria *E. coli*, *S. aureus*, and MRSA in the planktonic form before the biofilm formation. The obtained results are shown in Figure 2. The CV staining images showed that PtI and *Ec*-PtI+αA significantly reduced the *E. coli*, *S. aureus*, and *MRSA* biofilm formation (Figure S3, supplementary information). The discoloration was evident in PtI and *Ec*-PtI+αA compared to the control and αA-treated biofilms. Furthermore, the quantitative analysis of the prevention of biofilm was shown in Figure 2. The αA exhibited very low biofilm prevention of *E. coli*, *S. aureus*, and MRSA. The PtI caused 66, 64%, and 70% prevention of the biofilm of *E. coli*, *S. aureus*, and MRSA, respectively. However, *Ec*-PtI+αA exhibited 44%, 51%, and 77% prevention of the biofilm formation of *E. coli*, *S. aureus*, and MRSA, respectively. The obtained result indicated that PtI caused enhanced biofilm prevention in *E. coli* and *S. aureus* biofilms. However, the *Ec*-PtI+αA possessed enhancement in MRSA biofilm. Therefore, the PtI enzyme played a vital role in the prevention of all the biofilm formations. The *S. aureus*, *E. coli*, and MRSA biofilm matrixes contain protein components that maintain biofilm integrity [34,48]. The enzyme system of PtI and *Ec*-PtI+αA might be acted initially on bacterial adherence proteins and caused the prevention of biofilm formation. Proteinase and trypsin have frequently been used as efficient biofilm prevention agents that hinder bacterial adherence and biofilm formation in *S. aureus* [48]. The PtI and *Ec*-PtI+αA exhibited significant biofilm inhibition as well as prevention effects, while αA gave the biofilm inhibitions but not prevention. The CV staining data for biofilm prevention was validated by the bacterial colony counting technique in Figure 3. The biofilm after enzyme treatment was mixed with peptone water and cultured on Petri plates. The obtained data are shown in Figure 3. The control and αA-treated biofilm samples showed a very high number of colonies compared to the PtI- and *Ec*-PtI+αA-treated biofilm samples in Figure 3. Thus, the obtained results of CV staining and colony counting corroborated the efficacy of the PtI and *Ec*-PtI+αA for biofilm prevention of *E. coli*, *S. aureus*, and MRSA.

### 3.3. Proposed Mechanisms for the Anti-Biofilm Effect

**Figure 4:**
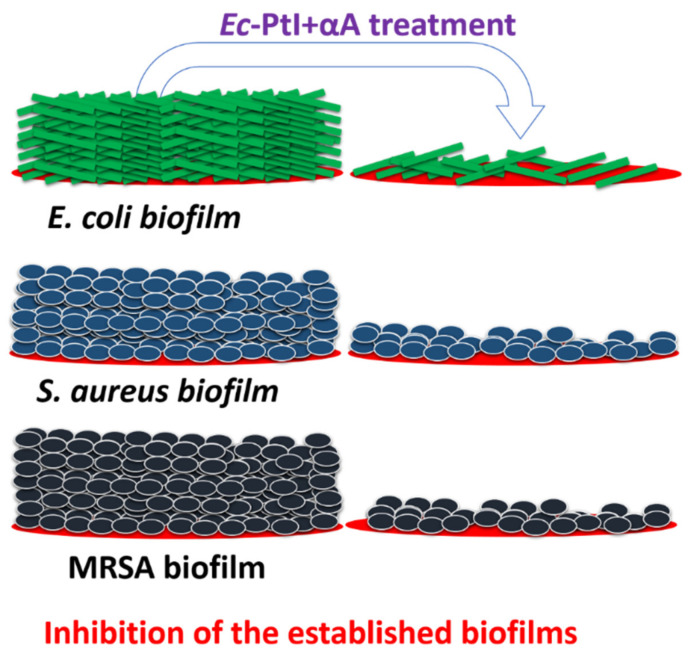
Schematic presentation of the inhibition of established biofilms.

The active PtI and αA acted on the backbone proteins of the EPS of established biofilms (Figure 4), this degraded the EPS backbone resulting in the respective biofilm eradication (Figure 4). In the enzymatic cocktail *Ec*-PtI+αA, PtI with αA acted on both proteins and starch components of EPS and elevated the biofilm eradication in MRSA biofilms (Figure 1).

**Figure 5:**
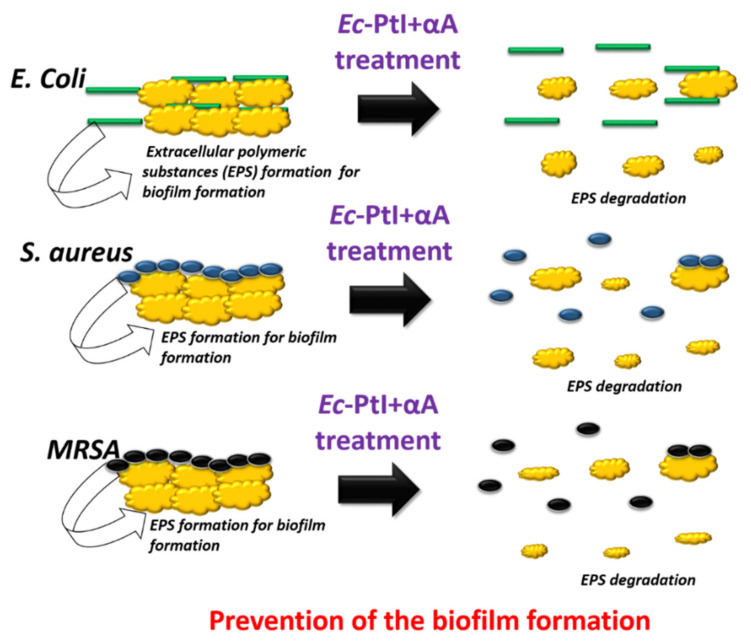
Schematic presentation of the prevention of the biofilm formation.

However, in the possible mechanism of the prevention effect, PtI acted on the proteins released from the planktonic cells for biofilm formation (Figure 5). This degradation of the proteins hampered the bacterial adherence processes and ultimately caused the prevention of biofilm formation (Figure 5). In the interesting observation of the prevention effect study, the individual αA exhibited a negligible biofilm prevention effect. This observation suggests that initially, proteins from EPS play an important role in the build-up of biofilm formation. Biofilm formation includes four main stages: bacteria attachment to surface, microcolony formation, biofilm maturation, and dispersal of bacterial biofilm [15]. The role of protein in the early stage of the bacterial attachment to the surface was investigated by Landini et al., 2010 [49]. Landini et al., 2010 concluded that initially proteins are involved in the bacterial cell attachment and then decipher the very early steps in biofilm formation [49]. The obtained results in our study are in line with this report. Hence, the degradation of the proteins at the early stage of biofilm formation might help the biofilm treatment significantly. 

After a close inspection of all the obtained results, it is very much clear that the different biofilms (*E. coli*, *S. aureus*, and MRSA) give a different response to αA, PtI, and *Ec*-PtI+αA enzyme systems. This is mainly due to the different scaffold and EPS composition of different bacteria [11]. Therefore, it is important to understand the EPS compositions in detail. The detailed composition of EPS of the *E. coli* biofilm was explained in [50]. The major proteins of the *E. coli* biofilm EPS are adhesins, these adhesins are transported to the extracellular environment by auto-transporter adhesins and explore the adhesin potential of *E. coli* [50]. Along with the expression of adhesins by *E. coli*, formation of biofilm matrix is vital for biofilm maturation. This biofilm matrix is an extremely complex, three dimensional background, and is fundamentally composed of water (97%), proteins, exopolysaccharide polymers, lipids/phospholipids, nucleic acids, metabolites, and absorbed nutrients [17,50,51,52,53]. The *S. aureus* biofilm EPS have mature *S. aureus* cells, primary oligosaccharide (polymer of *N*-acetyl-β-(1-6)-glucosamine), polysaccharide intercellular adhesin, eDNA, teichoic acids, secrete and lysis-derived proteins, accumulation-associated protein (Aap), and surfactant-like peptides (at the end of biofilm cycle for detachment) [54,55,56,57,58,59]. Thus, EPS of both the bacteria have a complex and three dimensional structure, and hence it is hard to quantify, as it varies greatly from species to species [14]. The adhesion extracellular proteins play a key role in complex EPS of both the biofilms. Therefore, breakdown of extracellularly secreted adhesins by PtI might be a key factor for the obtained anti-biofilm results for *E. coli* and *S. aureus*. The *E. coli* and *S. aureus* anti-biofilm effect observed in the presence of αA is less than that for PtI, and it is suggested that breakdown of the proteins than polysaccharides from EPS elevates the anti-biofilm effect. However, the combination of enzymes *Ec*-PtI+αA helped to encounter MRSA biofilm EPS, and hence degrading polysaccharides and proteins at the same time elevated the anti-biofilm effect in MRSA compared with the individual αA and PtI. 

Earlier PtI was reported for the extraction of the hemicellulose from wheat germs [60]. The bovine pancreas is a rich source of many therapeutic enzymes [61]. The major function of the bovine pancreatic acinar cell is the synthesis, storage, and secretion of several digestive enzymes, such as proteases, amylase, lipase, elastase, and ribonucleases, to catalyze the food constituent hydrolysis into absorbable forms [30]. This is mostly applied in human therapeutic applications [28,29,31,62]. In line with that, our study exploits the PtI from the bovine pancreas for the anti-biofilm properties. Our study showed the PtI encompasses excellent anti-biofilm properties against both “drug-resistant MRSA”, and non-drug resistant pathogenic *E. coli* and *S. aureus*, biofilms. As the pancreatic enzyme is a well-known source of therapeutic enzymes, PtI as a potent anti-biofilm enzyme might be valuable in future studies.

### 3.4. Anti-Bacterial Assessment of the Enzymes αA, PtI, and Ec-PtI+αA

**Figure 6:**
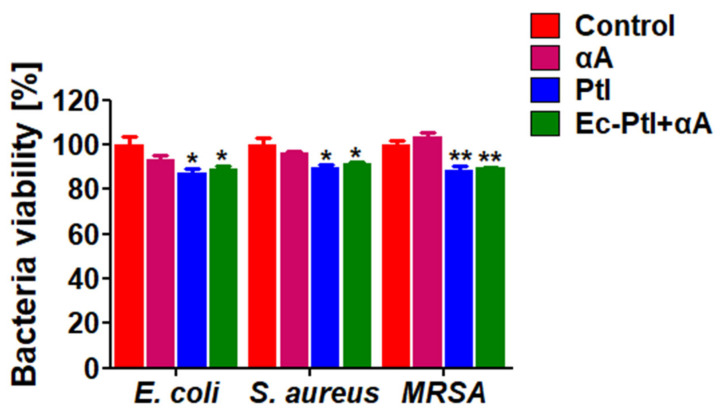
Anti-bacteria effect treated with protease and co-treatment with α-amylase against *E. coli*, *S. aureus*, and MRSA, respectively. All data were expressed as the mean ± SEM (*n* = 3) * *p* < 0.05 and ** *p* < 0.01 vs. control.

After assessing the biofilm inhibition and prevention activities, it is very important to test the anti-bacterial effect exerted by αA, PtI, and *Ec*-PtI+αA on *E. coli*, *S. aureus*, and MRSA. Hence, the αA, PtI, and *Ec*-PtI+αA was tested for anti-bacterial studies (Figure 6). The obtained data in Figure 6 conveyed that αA, PtI, and *Ec*-PtI+αA do not inhibit the bacterial growth significantly in *E. coli*, *S. aureus*, and MRSA. However, a very low 5–7% inhibition was observed in the PtI- and *Ec*-PtI+αA-treated samples. This might be due to the enzyme PtI. There are several proteases which have anti-microbial properties [63,64]. The mechanism of the proteinase inhibited growth of the *Fusarium solani* and *Staphylococcus aureus* and was explained as the alteration of cell plasma membrane by protease activity [63]. A similar mechanism of membrane alteration might have occurred with PtI-mediated very low anti-microbial activity. The overall results validated the not significant anti-bacterial nature of the studied enzymes αA, PtI, and *Ec*-PtI+αA. Hence, our study recommends, the individual enzymes PtI and *Ec*-PtI+αA are mainly used to combat the most daunting task of biofilm dispersal. After the dispersal of the bacteria, it can easily be killed by either antibiotics or nano-formulations. Futhermore, it may be further possible to make a combination of these enzymes with the anti-microbial compounds to achieve both the goal of dispersal and killing of the bacteria at the same time.

## 4. Conclusions

In summary, the enzyme PtI and its combinations with αA were assessed for anti-biofilm activities (inhibition of established biofilms and prevention effect on biofilm formation) against three major biofilms of *E. coli*, *S. aureus*, and MRSA. The PtI showed excellent anti-biofilm activities both in biofilm inhibition and prevention against the *E. coli*, *S. aureus*, and MRSA biofilms. This study marks the importance of PtI for future anti-biofilm treatments. In the future, PtI can be coupled with potent anti-microbial masteries to enhance biofilm treatment. Thus, PtI and its combination with αA can be used as an excellent treatment approach for biofilm dispersal. These enzymatic assessments might be extremely helpful in the development of the future treatment of biofilm infection. 

## Figures and Tables

**Table 1:** The detailed account of several reported protease for biofilm inhibition.

Enzyme	Sources	Biofilms Inhibition (%)	Target Bacteria	Reference
PtI	Bovine pancreas	87	*E. coli*	This work
		89	*S. aureus*	
		67	MRSA	
Flavourzyme	*Aspergillus oryzae*	50	*S. epidermidis*	[39]
Neutrase	*Bacillus amyloliquefaciens*	72	*S. aureus*	[39]
		35	*S. epidermidis*	
Alcalase	*B. licheniformis*	25	*S. epidermidis*	[39]
α-amylase	*B. subtilis*	50	*Pseudomonas aeruginosa*	[46]
		65	*Vibrio cholerae*	
		70	MRSA	
Aureolysin	*S. aureus*	50	*S. aureus*	[38]
		33	*S. epidermidis*	
Dispersin B	*Aggregatibacter* *actinomycetemcomitans*	50	*S. epidermidis*	[38]
Proteinase K	*Tritirachium album*	5	*Pseudomonas aeruginosa*	[47]
		10	*Vibrio cholerae*	
		5	MRSA	
		75	*S. aureus*	
		90	*L. monocytogenes*	
Papain	Papaya	80	*L. monocytogenes*	[32]
Trypsin	PA clan superfamily	20	*Pseudomonas aeruginosa*	[46]

## Supplementary Materials

The following are available online at https://www.mdpi.com/2073-4360/12/12/3032/s1. Figure S1. Inhibition effect of PtI and *Ec*-PtI+αA against biofilm was performed by crystal violet (CV) staining. Figure S2. Inhibition effect of protease and protease with α- amylase against *E. coli*, *S. aureus*, MRSA in biofilm were performed by colony counting. Figure S3. Prevention effect of PtI, αA, and *Ec*-PtI+αA against biofilm was performed by crystal violet (CV) staining.
