# Supplementary material for: Efficient Biofilms Eradication by Enzymatic-Cocktail of Pancreatic Protease Type-I and Bacterial α-Amylase
Source: Polymers (Basel). 2020 Dec 17;12(12):3032. doi: 10.3390/polym12123032 (PMC7766206; doi:10.3390/polym12123032)
Supplement: Supplementary file 1 [file polymers-12-03032-s001.pdf]

Supplementary Material

Efficient Biofilms Eradication by Enzymatic-cocktail of Pancreatic Protease Type-I and Bacterial α-Amylase

Seung-Cheol Jee [1], Min Kim [1], Jung-Suk Sung [1] and Avinash A. Kadam [2,*]

[1] Department of Life Science, College of Life Science and Biotechnology, Dongguk University-Seoul, Biomedi Campus, 32 Dongguk-ro, Ilsandong-gu, Goyang-si 10326, Gyeonggi-do, South Korea

[2] Research Institute of Biotechnology and Medical Converged Science, Dongguk University-Seoul, Biomedi Campus, 32 Dongguk-ro, Ilsandong-gu, Goyang-si 10326, Gyeonggi-do, South Korea

* Correspondence: avikadam2010@gmail.com; kadamavinash@dongguk.edu, Tel.: 82-31-961-5616; Fax.: 82-31-961-5108

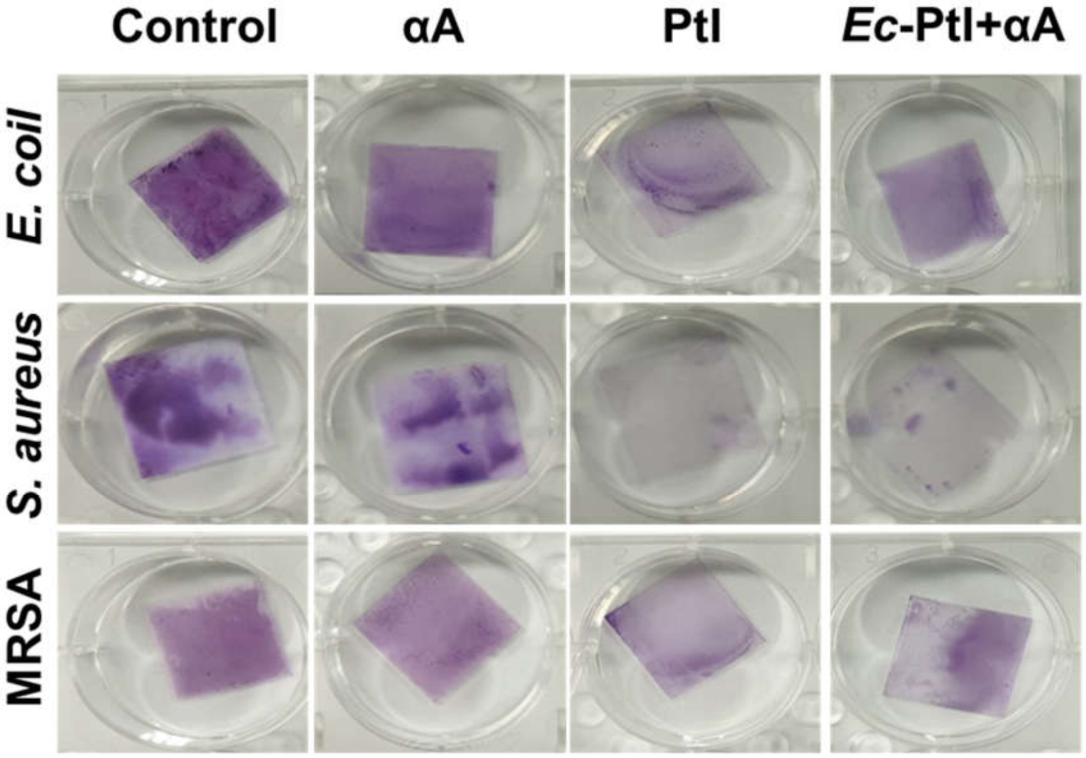

Figure S1. Inhibition effect of PtI and *Ec*-PtI+αA against biofilm was performed by crystal violet (CV) staining.

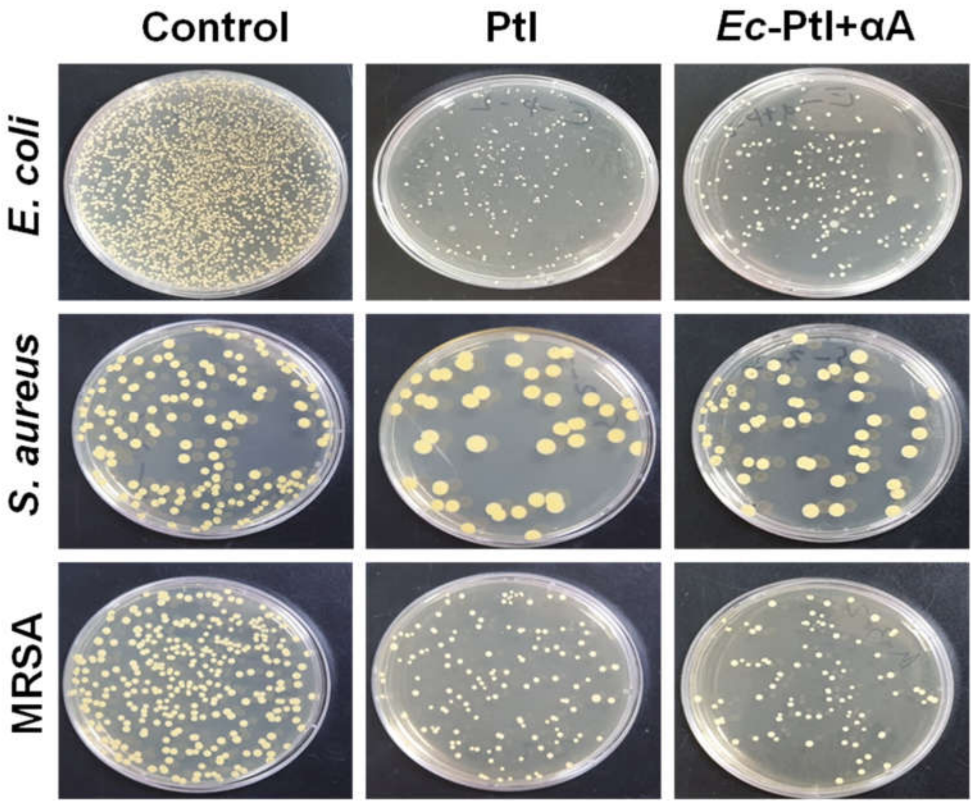

Figure S2. Inhibition effect of protease and protease with α- amylase against *E. coli*, *S. aureus*, MRSA in biofilm were performed by colony counting.

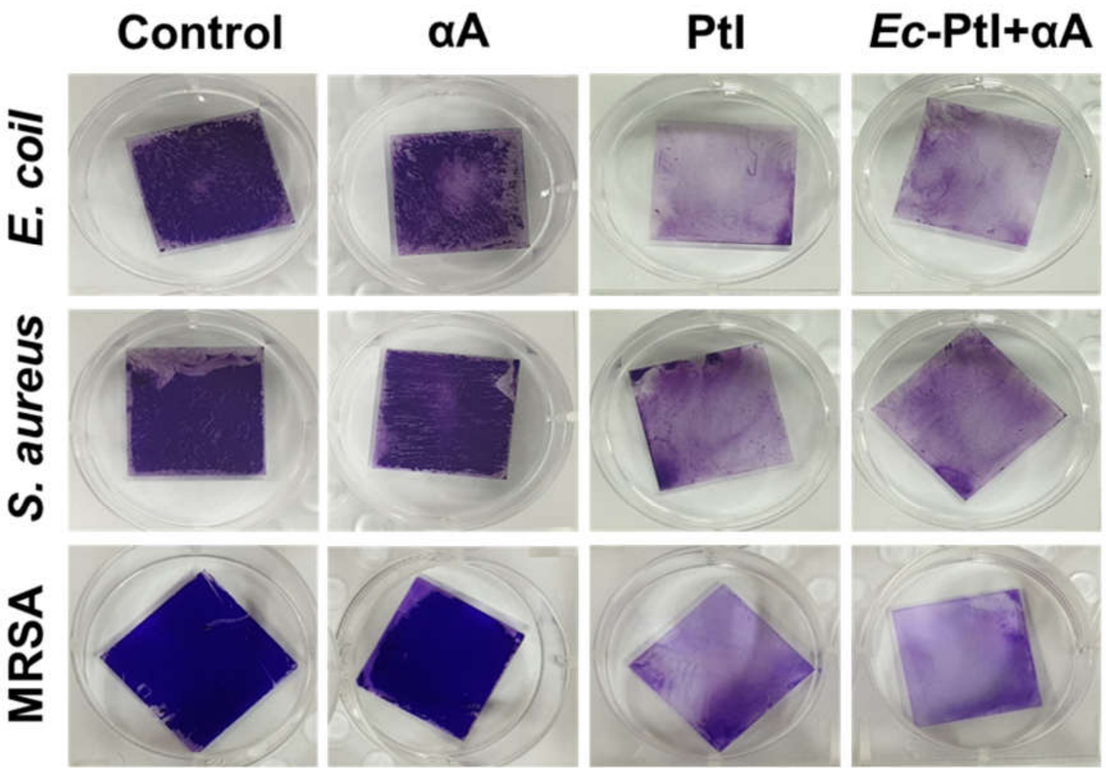

Figure S3. Prevention effect of PtI, αA, and Ec-PtI+αA against biofilm was performed by crystal violet (CV) staining.
